# Improvements in lung function following vitamin C supplementation to pregnant smokers are associated with buccal DNA methylation at 5 years of age

**DOI:** 10.1186/s13148-024-01644-8

**Published:** 2024-02-27

**Authors:** Lyndsey E. Shorey-Kendrick, Cindy T. McEvoy, Kristin Milner, Julia Harris, Julie Brownsberger, Robert S. Tepper, Byung Park, Lina Gao, Annette Vu, Cynthia D. Morris, Eliot R. Spindel

**Affiliations:** 1 Division of Neuroscience, Oregon National Primate Research Center, Oregon Health and Science University, Beaverton, OR 97006 USA; 2 Department of Pediatrics, Pape Pediatric Research Institute, Oregon Health and Science University, Portland, OR USA; 3 Department of Pediatrics, Oregon Health and Science University, Portland, OR USA; 4 Department of Pediatrics, Herman B Wells Center for Pediatric Research, Indiana University School of Medicine, Indianapolis, IN USA; 5 Biostatistics Shared Resources, Knight Cancer Institute, Bioinformatics and Biostatistics Core, Oregon National Primate Research Center, Oregon Health and Science University, Portland State University School of Public Health, Portland, OR USA; 6 Oregon Clinical & Translational Research Institute, Oregon Health and Science; Department of Medical Informatics and Clinical Epidemiology, Oregon Health and Science University, Portland, OR USA

**Keywords:** MSDP: maternal smoking during pregnancy, Vitamin C, RCT: randomized clinical trial, DNA methylation, MethylationEPIC, Nicotine, Lung function, Airway, Wheeze

## Abstract

**Background:**

We previously reported in the “Vitamin C to Decrease the Effects of Smoking in Pregnancy on Infant Lung Function” randomized clinical trial (RCT) that vitamin C (500 mg/day) supplementation to pregnant smokers is associated with improved respiratory outcomes that persist through 5 years of age. The objective of this study was to assess whether buccal cell DNA methylation (DNAm), as a surrogate for airway epithelium, is associated with vitamin C supplementation, improved lung function, and decreased occurrence of wheeze.

**Methods:**

We conducted epigenome-wide association studies (EWAS) using Infinium MethylationEPIC arrays and buccal DNAm from 158 subjects (80 placebo; 78 vitamin C) with pulmonary function testing (PFT) performed at the 5-year visit. EWAS were performed on (1) vitamin C treatment, (2) forced expiratory flow between 25 and 75% of expired volume (FEF_25–75_), and (3) offspring wheeze. Models were adjusted for sex, race, study site, gestational age at randomization (≤ OR > 18 weeks), proportion of epithelial cells, and latent covariates in addition to child length at PFT in EWAS for FEF_25–75_. We considered FDR *p* < 0.05 as genome-wide significant and nominal *p* < 0.001 as candidates for downstream analyses. Buccal DNAm measured in a subset of subjects at birth and near 1 year of age was used to determine whether DNAm signatures originated in utero, or emerged with age.

**Results:**

Vitamin C treatment was associated with 457 FDR significant (*q* < 0.05) differentially methylated CpGs (DMCs; 236 hypermethylated; 221 hypomethylated) and 53 differentially methylated regions (DMRs; 26 hyper; 27 hypo) at 5 years of age. FEF_25–75_ was associated with one FDR significant DMC (cg05814800), 1,468 candidate DMCs (*p* < 0.001), and 44 DMRs. Current wheeze was associated with 0 FDR-DMCs, 782 candidate DMCs, and 19 DMRs (*p* < 0.001). In 365/457 vitamin C FDR significant DMCs at 5 years of age, there was no significant interaction between time and treatment.

**Conclusions:**

Vitamin C supplementation to pregnant smokers is associated with buccal DNA methylation in offspring at 5 years of age, and most methylation signatures appear to be persistent from the prenatal period. Buccal methylation at 5 years was also associated with current lung function and occurrence of wheeze, and these functionally associated loci are enriched for vitamin C associated loci.

*Clinical trial registration* ClinicalTrials.gov, NCT01723696 and NCT03203603.

**Supplementary Information:**

The online version contains supplementary material available at 10.1186/s13148-024-01644-8.

## Background

In utero exposure to maternal cigarette smoking during pregnancy (MSDP) is a well-established risk factor for prematurity, intrauterine growth restriction (IUGR), and perinatal death [1–5]. Longitudinal studies demonstrate that airway function trajectories are established in early life and that children in lower airway function percentiles are more likely to have lower airway function in adulthood [6, 7]. Therefore, offspring exposed to MSDP exhibit lifetime decreases in airway function and increased risk for wheeze and asthma [8–10]. However, despite smoking cessation efforts, ~ 8% of women in the USA continue to smoke in pregnancy, resulting in more than 400,000 babies exposed annually to MSDP [11, 12]. Given the significant negative impact that MSDP has on lifelong respiratory morbidity, our group has investigated interventions to mitigate these effects in offspring unwillingly exposed to in utero smoke. Following preclinical evidence in non-human primate studies [13], we have demonstrated in two randomized controlled trial (RCT) populations that vitamin C supplementation to pregnant smokers can improve airway function and reduce the occurrence of wheeze [14, 15]. In our second RCT, these improvements in lung function and the occurrence of wheeze with vitamin C supplementation are persistent to at least 5 years of age [16].

The epigenome, and particularly DNA methylation (DNAm), is responsive to environmental exposures, and is a mechanism for regulation of gene expression [17]. Modification of DNAm during embryonic and fetal development may lead to structural and functional alterations in the developing lung and/or predispose risk for respiratory infections later in life through changes in the immune response. Exposure to MSDP is associated with altered DNA methylation in placenta, blood, and buccal epithelium [18–29], and may provide a mechanistic link between in utero exposures and future health outcomes [30–33]. Previous studies have reported DNAm as a mediator of the effects of MSDP on health outcomes such as reduced birth weight and psychiatric morbidity [18, 28, 34]. In our pilot clinical trial population, we demonstrated using targeted bisulfite sequencing that vitamin C supplementation during pregnancy could restore levels of DNAm in candidate genes in placenta, cord blood, and childhood buccal DNA in parallel with improved lung function [35]. More recently, we performed epigenome-wide analysis of placental DNAm in a subset of participants from our second RCT and showed that vitamin C supplementation prevented or restored some of the effects of MSDP on DNAm, in association with improvements in placental function and respiratory outcomes [36].

Gene expression changes in bronchial epithelium in response to cigarette smoke are reflected in buccal epithelium [37]. Similarly, DNAm profiles in buccal epithelium are a proxy of DNAm profiles in airway epithelium, and buccal swabs are minimally invasive to collect [38]. In the current study, we aimed to investigate whether DNAm signatures in buccal epithelium collected at 5 years of age differed between offspring whose mothers were randomized to vitamin C versus placebo during pregnancy. We hypothesized that buccal DNA profiles would be different between RCT groups and be directly associated with respiratory outcomes in the same subjects, which may provide mechanistic insight for the observed effect of persistently improved lung function.

To test this hypothesis, we measured buccal DNAm genome-wide using the Illumina MethylationEPIC array platform in 158 subjects with both pulmonary function testing by spirometry and buccal DNA collection at the 5-year-old follow-up visit [16]. We identified differentially methylated CpGs (DMCs) and differentially methylated regions (DMRs) between vitamin C and placebo groups. Additionally, we performed epigenome-wide association studies (EWAS) of forced expiratory flow at mid-expiration (forced expiratory flow between 25 and 75% expired volume; FEF_25–75_) and of current wheeze. We compared our results with those from previous EWAS and performed mediation analyses to provide additional biological support for our findings. Lastly, in a subset of 37 participants with buccal DNAm collected near birth and near 12 months of age, we investigated whether these signatures originated in utero and were persistent, or emerging with age.

## Results

### Patient demographics

Out of 243 offspring delivered as part of our VCSIP RCT, 213 were re-consented into follow-up through 5 years of age, and 192 performed technically acceptable spirometry at 5 years of age. Of those 192 subjects, 158 had sufficient quality buccal DNA for epigenome-wide methylation analysis (*n* = 80 placebo, 78 vitamin C; Additional file 1: Fig. 1). Table 1 summarizes the demographics, tobacco exposure, and respiratory outcomes for these 158 subjects by group. There were no significant differences between the vitamin C and placebo groups for maternal demographics, infant/child demographics, or levels of prenatal or postnatal smoke exposure. At the randomization visit, maternal vitamin C levels were not different between groups, and increased significantly after randomization only in the vitamin C group. At the 5-year follow-up visit, there was no difference between groups in child length; however, measurements of forced expiratory flows by spirometry were significantly greater in the vitamin C group, and the occurrence of wheeze at 4–6 years of age was lower as previously published [16].

**Table 1:** Demographics of subjects included in analysis

	*n* missing	Placebo (*n* = 80)	Vitamin C (*n* = 78)	*p* value*
*Maternal variables*				
Age at enrollment (mean (SD))		27.11 (6.26)	26.62 (5.28)	0.591
*Education level *(%)				0.205
Less than high school		19 (23.8)	14 (17.9)	
High school degree/ GED		25 (31.2)	37 (47.4)	
Some college		32 (40.0)	25 (32.1)	
Bachelor’s degree (BA, BS)		4 (5.0)	2 (2.6)	
BMI at enrollment (mean (SD))		30.45 (7.68)	28.84 (6.57)	0.16
Gravida (mean (SD))		3.55 (2.38)	3.29 (2.19)	0.485
Maternal asthma (%)		26 (32.5)	26 (33.3)	1
*Study variables*				
Site (%)				0.93
OHSU		13 (16.2)	13 (16.7)	
SWW		37 (46.2)	38 (48.7)	
IA		30 (37.5)	27 (34.6)	
GA at randomization > 18 weeks (%)		40 (50.0)	48 (61.5)	0.194
*Plasma vitamin C in pregnancy*				
Randomization (mean (SD))	13	47.59 (17.65)	48.18 (20.18)	0.848
Mid-gestation (mean (SD))	8	39.71 (19.04)	59.38 (23.35)	** < 0.001**
Late-gestation (mean (SD))	29	37.87 (15.27)	51.62 (22.48)	** < 0.001**
*Smoking variables*				
Cigarettes per day (mean (SD))		8.06 (5.12)	7.59 (4.53)	0.54
Pack yrs (mean (SD))		11.24 (9.46)	10.63 (8.31)	0.671
Plasma cotinine (ng/ml)				
Randomization (mean (SD))	17	67.87 (44.97)	83.73 (56.41)	0.061
Mid-gestation (mean (SD))	12	75.04 (126.91)	68.60 (51.20)	0.682
Late-gestation (mean (SD))	38	61.80 (44.57)	61.18 (36.48)	0.93
*Maternal hair nicotine (ng/mg)*				
Randomization (median [IQR])	19	2.68 [0.68, 6.39]	3.20 [1.27, 9.84]	0.171
Delivery (median [IQR])	35	1.50 [0.71, 5.57]	2.14 [1.23, 4.60]	0.239
Postpartum 3 months (median [IQR])	14	3.41 [1.03, 7.90]	3.30 [1.25, 8.07]	0.807
Postpartum 12 months (median [IQR])	30	4.58 [1.58, 9.79]	4.72 [2.01, 9.53]	0.551
Postpartum 48 months (median [IQR])	24	1.29 [0.34, 3.97]	2.10 [0.67, 3.92]	0.125
Postpartum 60 months (median [IQR])	14	1.85 [0.41, 4.30]	1.82 [0.67, 3.43]	0.588
*Child hair nicotine (ng/mg)*				
Delivery (median [IQR])	85	0.37 [0.17, 0.69]	0.30 [0.18, 0.73]	0.973
Postnatal 3 months	21	2.54 [1.63, 6.64]	3.64 [2.17, 6.89]	0.155
Postnatal 12 months	23	1.60 [0.74, 4.53]	2.06 [1.02, 4.07]	0.205
Postnatal 48 months	15	0.33 [0.14, 1.80]	0.58 [0.13, 1.53]	0.638
Postnatal 60 months	8	0.34 [0.08, 1.18]	0.35 [0.13, 0.79]	0.86
*Infant variables*				
GA at birth (mean (SD))		38.61 (1.63)	38.73 (1.83)	0.685
Preterm (%)		4 (5.0)	9 (11.5)	0.228
White (%)		65 (81.2)	59 (75.6)	0.507
Sex = Female (%)		42 (52.5)	36 (46.2)	0.523
*Child variables at 5YO Visit*				
PFT height (mean (SD))		113.15 (5.78)	112.52 (4.88)	0.456
FEF_25–75_(L/sec) (mean (SD))		1.28 (0.31)	1.47 (0.37)	** < 0.001**
FEF_50_ (L/sec) (mean (SD))		1.44 (0.34)	1.62 (0.38)	**0.002**
FEF_75_ (L/sec) (mean (SD))		0.64 (0.19)	0.79 (0.26)	** < 0.001**
FEV_1_ (L/sec) (mean (SD))		1.13 (0.19)	1.15 (0.19)	0.403
FEV_1_/FVC (mean (SD))		86.56 (5.77)	90.15 (6.70)	** < 0.001**
Wheeze at 4–6 yrs of age = Yes (%)		39 (48.8)	22 (28.2)	**0.013**

^*^P values calculated by Chi-square test for categorical data, by Fisher’s exact test for normally distributed variables, and by Wilcox test for non-normally distributed variables

### EWAS of vitamin C vs placebo in buccal DNAm at 5 years

**Fig. 1:**
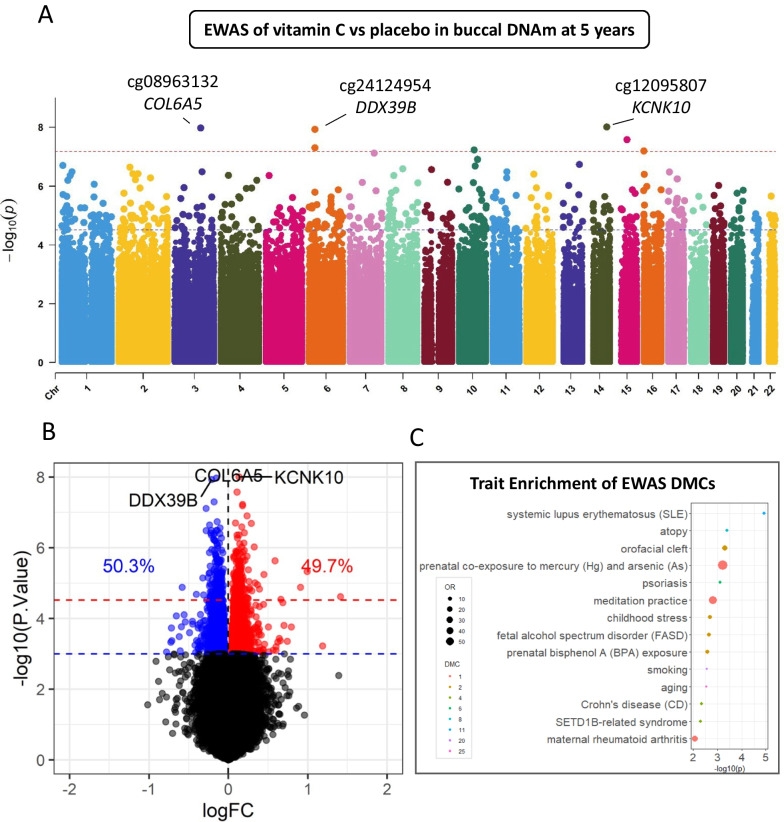
Summary of EWAS of vitamin C vs placebo in buccal DNAm and 5 years. The Manhattan plot **A** shows the –log10(P) on the y-axis and chromosome position on the x-axis; red line indicates Bonferroni adjusted *p* value < 0.05 and blue line indicates FDR *p* < 0.05; labels annotate the top 3 DMCs. The volcano plot **B** shows the –log10(*P* value) on the y-axis and effect size (logFC) on the x-axis. Hypermethylated CpGs (i.e., higher methylation level in participants with vitamin C compared to those with placebo) are shown in red; hypomethylated CpGs in blue. The red line indicates FDR adjusted *p* value < 0.05 and blue line indicates candidates with nominal *p* < 0.001. The dot plot **C** shows enrichment of traits previously associated with the differentially methylated CpGs identified in this study following prenatal vitamin C supplementation

We examined whether DNAm profiles in buccal epithelium collected at 5 years of age differed between offspring of pregnant smokers randomized to vitamin C versus placebo using 746,421 CpGs on the Illumina EPIC array that passed QC. Our CpG-specific models were adjusted for covariates of sex, race, study site, gestational age at randomization (≤ OR > 18 weeks), proportion of epithelial cells, and latent covariates. Vitamin C treatment was associated with 457 DMCs at FDR significance (236 hypermethylated; 221 hypomethylated), annotated to 438 unique genes (Additional file 2: Table S1), and 5,379 putative DMCs at *p* < 0.001. The top 20 FDR significant DMCs associated with prenatal vitamin C at 5 years are shown in Table 2. The top 3 vitamin C treatment DMCs were annotated to potassium two pore domain channel subfamily K member 10 (*KCNK10*; cg12095807; logFC = 0.135), collagen type VI alpha 5 chain (*COL6A5*; cg08963132; logFC = − 0.152), and DExD-box helicase 39B (*DDX39B*; cg24124954; logFC = − 0.209) genes (Fig. 1A, B). In a sensitivity analysis without adjustment for cell composition and latent covariates, cg12095807 and cg08963132 remained FDR significant, while cg24124954 was no longer FDR significant but had a similar direction and magnitude of effect.

**Table 2:** Top 20 DMCs associated with vitamin C supplementation

Probe	CHR	MAPINFO	Feature-CpGisland	Nearest genes	Direction	logFC	P.Value	Adjusted P.Value
cg12095807	14	88,700,366	Body-opensea	*KCNK10*	HYPER	0.134	9.87E−09	2.96E−03
cg08963132	3	130,064,261	TSS200-shore	*COL6A5*	HYPO	− 0.152	1.07E−08	2.96E−03
cg24124954	6	31,508,106	Body-shore	*DDX39B*	HYPO	− 0.210	1.19E−08	2.96E−03
cg06781424	15	51,369,527	Body-opensea	*TNFAIP8L3*	HYPER	0.108	2.67E−08	4.98E−03
cg20804756	6	31,507,857	Body-shore	*DDX39B*	HYPO	− 0.181	5.00E−08	6.87E−03
cg00813090	10	75,386,088	IGR-shore	*MYOZ1*	HYPER	0.178	5.93E−08	6.87E−03
cg15948509	16	1,832,913	TSS200-island	*NUBP2*	HYPER	0.177	6.45E−08	6.87E−03
cg05522700	7	120,589,681	TSS1500-shore	*ING3*	HYPO	− 0.281	7.68E−08	7.17E−03
cg01227964	10	92,683,897	IGR-opensea	*ANKRD1*	HYPER	0.234	1.24E−07	1.03E−02
cg10758716	13	99,184,827	Body-opensea	*STK24*	HYPO	− 0.140	1.82E−07	1.21E−02
cg01803717	1	8,939,106	TSS1500-island	*ENO1*	HYPER	0.089	1.99E−07	1.21E−02
cg22378064	10	80,476,116	IGR-opensea	*LINC00856*	HYPER	0.290	2.06E−07	1.21E−02
cg09585004	2	56,236,485	Body-opensea	*CCDC85A*	HYPO	− 0.236	2.27E−07	1.21E−02
cg11878331	8	72,469,507	IGR-island	*EYA1*	HYPER	0.167	2.57E−07	1.21E−02
cg13392861	9	37,914,861	IGR-opensea	*SHB*	HYPO	− 0.103	2.76E−07	1.21E−02
cg24067803	11	70,653,378	Body-opensea	*SHANK2*	HYPO	− 0.138	3.23E−07	1.21E−02
cg12691620	1	53,152,081	3'UTR-opensea	*COA7*	HYPO	− 0.214	3.24E−07	1.21E−02
cg17995273	3	136,936,856	IGR-opensea	*IL20RB*	HYPO	− 0.154	3.25E−07	1.21E−02
cg10693616	17	7,982,670	Body-island	*ALOX12B*	HYPO	− 0.280	3.28E−07	1.21E−02
cg01109169	2	71,134,630	Body-island	*VAX2*	HYPER	0.201	3.78E−07	1.21E−02

Differentially methylated region (DMR) analysis identified 53 FDR significant regions (26 hyper; 27 hypo) associated with vitamin C supplementation during pregnancy in the buccal DNA at 5 years of age. The top 10 vitamin C associated DMRs are shown in Table 3 and the full list in Additional file 2: Table S2. The three most significant DMRs were all hypomethylated and were annotated to *DDX39B* (DExD-box helicase 39B; 14 CpGs; meandiff = − 0.008), *FXYD1* (FXYD domain containing ion transport regulator 1; 17 CpGs; meandiff = − 0.013), and *NDUFAF3* (NADH: ubiquinone oxidoreductase complex assembly factor 3; 14 CpGs; meandiff =− 0.007). The next most significant DMRs were hypermethylated and annotated to *VAX2* (ventral anterior homeobox 2; 8 CpGs; meandiff = 0.013) and *DDO* (D-aspartate oxidase; 6 CpGs; meandiff = 0.036).

**Table 3:** Top 10 DMRs associated with vitamin C supplementation

Seqnames	Start	End	Width	# CpGs	Min_smoothed_fdr	Direction	Maxdiff	Meandiff	Overlapping.genes
chr6	31,507,316	31,508,665	1350	14	6.77E−22	HYPO	− 0.020	− 0.008	*DDX39B, ATP6V1G2-DDX39B*
chr19	35,629,273	35,630,651	1379	17	7.24E−19	HYPO	− 0.027	-0.013	*FXYD1, CTD-2527I21.4, LGI4, AC020907.2*
chr3	49,057,259	49,058,430	1172	14	1.03E−18	HYPO	− 0.015	− 0.007	*NDUFAF3, DALRD3, MIR425, MIR191*
chr2	71,133,854	71,134,936	1083	8	2.11E−15	HYPER	0.021	0.013	*VAX2*
chr6	110,720,501	110,721,629	1129	6	4.63E−15	HYPER	0.061	0.036	*DDO*
chr8	38,757,532	38,757,859	328	5	9.17E−15	HYPO	− 0.036	− 0.024	*NA*
chr11	66,233,183	66,234,186	1004	12	1.39E−13	HYPO	− 0.026	− 0.007	*MRPL11*
chr17	42,081,179	42,082,647	1469	13	5.25E−12	HYPO	− 0.022	− 0.006	*NAGS, PYY*
chr11	2,153,991	2,154,952	962	10	2.62E−11	HYPER	0.026	0.014	*IGF2, INS-IGF2*
chr20	57,426,757	57,427,650	894	29	2.89E−11	HYPO	− 0.036	0.006	*GNAS*

### Comparison of vitamin C DMCs with previous EWAS

We investigated whether the CpGs associated with vitamin C supplementation during pregnancy in this study have been associated with traits related to airway function or related diseases in previous EWAS. We used the EWAS Atlas knowledgebase to download curated tables of EWAS associations from over 1000 publications that includes 729 traits and 643,805 significant CpG-trait associations [39]. From our list of 457 FDR significant vitamin C treatment DMCs, 165 were previously associated with one or more traits from this database, and 112 unique traits mapped to one or more DMCs (Additional file 2: Table S3). Vitamin C DMCs were significantly enriched for CpGs previously associated with 14 unique traits, with systemic lupus erythematosus (OR = 3.00; *p* = 1.19e−05) as the most enriched trait (Fig. 1C). Atopy was the second most enriched trait (OR = 2.1; *p* = 3.75Ee−02) with eight vitamin C DMCs in this study previously associated with atopy in nasal epithelium [40]. Several additional traits related to immune function were enriched for treatment DMCs, including psoriasis, atopic dermatitis, Crohn’s disease, and maternal rheumatoid arthritis **(**Fig. 1C**)**. Additionally, DMCs were enriched for CpGs previously associated with smoking, and one of the top vitamin C DMCs in this study (cg24124954) annotated to the *DDX39B* gene was associated with six EWAS traits including maternal smoking, alcohol consumption, and adult smoking **(**Additional file 2: Table S3**)**.

### EWAS of lung function at 5 years of age

We previously published that offspring born to vitamin C supplemented pregnant smokers had significantly increased forced expiratory flows (FEFs) relative to placebo subjects at 5 years of age [16]. We therefore examined association of buccal DNAm with lung function (specifically FEF_25–75_) measured at the same 5-year-old study visit. Our CpG-specific models were adjusted for covariates of length at PFT, sex, race, study site, gestational age at randomization (≤ OR > 18 weeks), proportion of epithelial cells, and latent covariates. One CpG (cg05814800) annotated to *FAM181A* (family with sequence similarity 181 member A) reached FDR significance after multiple testing correction. Using a threshold of *p* = 0.001 to identify candidates for downstream analysis, 1,468 putative DMCs were associated with FEF_25–75_ **(**Additional file 2: Table S4). We also identified 44 candidate DMRs associated with FEF_25–75_ (Additional file 2: Table S5). The top DMR associated with FEF_25–75_ (min smoothed fdr = 2.71e−28) spanned 335 bp in an intergenic region upstream of the *POU5F1* (POU class 5 homeobox 1) gene and *PSORS1C3* (psoriasis susceptibility 1 candidate 3 non-coding RNA) and contained 14 CpGs, all negatively associated with FEF_25–75_. The next FEF_25–75_ candidate DMR was annotated to *FAM181A,* spanned 1,099 bp and contained 12 CpGs (min smoothed fdr = 1.91e-14; hypermethylated).

### EWAS of current wheeze at 5 years of age

A secondary outcome in our randomized clinical trial of supplemental vitamin C versus placebo to pregnant smokers was the occurrence of wheeze. We measured a significant decrease in wheeze in the offspring of pregnant smokers randomized to vitamin C versus placebo (28.3% vs 47.2%; estimated odds ratio, 0.41 [95% CI, 0.23–0.74]; *P* = 0.003) [16]. Association of the occurrence of wheeze at 4 to 6 years of age with buccal DNAm at 5 years of age did not result in any FDR significant DMCs after adjusting for sex, race, study site, gestational age at randomization (≤ OR > 18 weeks), proportion of epithelial cells, and latent covariates. Using a less stringent threshold of *p* = 0.001 to identify candidates for downstream analysis, we identified 782 putative DMCs and 19 putative DMRs (Additional file 2: Tables S6 and S7). The top putative DMC associated with wheeze at 4–6 years of age was annotated to *ARHGAP26* (Rho GTPase activating protein 26), and the top candidate DMR was annotated to *GSTT1* (glutathione S-transferase theta 1) and spanned a region of 840 bp containing 11 CpGs hypermethylated in subjects with wheeze (min smoothed fdr = 3.22e−16).

### DNA methylation mediates some of the vitamin C treatment effect on lung function

**Fig. 2:**
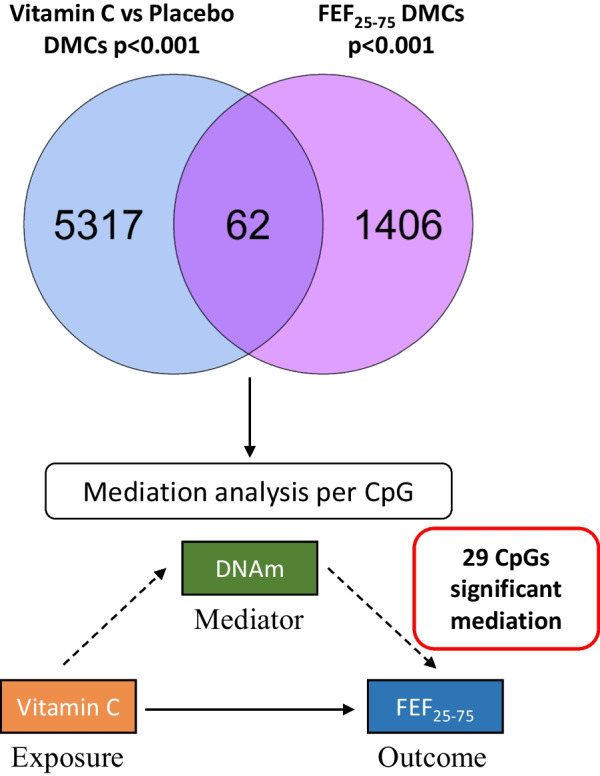
Overlap of vitamin C DMCs with lung function DMCs and mediation analysis. The Venn diagram (top) shows the overlap of 62 CpGs associated with vitamin C supplementation (in blue) and FEF_25–75_ (in pink) both at *p* < 0.001. Mediation analysis was performed for each of the 62 CpGs associated with both exposure and outcome as indicated in the schematic

We next examined the overlap of probes differentially methylated with lung function and/or wheeze (*p* < 0.001) with those associated with vitamin C treatment, and tested whether the overlap was significant using the hypergeometric test. Out of 1468 candidate CpGs associated with FEF_25–75_, 62 were also associated with vitamin C treatment at *p* < 0.001, more than expected by chance (*p* value = 1.6e−28), while only nine out of 782 candidate wheeze DMCs were also associated with vitamin C treatment (*p* = 0.06). In the 62 CpGs which overlapped between vitamin C treatment and FEF_25–75_ at the *p* < 0.001 threshold, we performed mediation analysis **(**Fig. 2**)** to test whether DNAm mediates any proportion of the association between vitamin C treatment and lung function, and found that 29 CpGs had an FDR adjusted *p* value less than 0.05 (Additional file 2: Table S8). The largest effect size was observed for cg12183021, annotated to *JAKMIP1* (janus kinase and microtubule interacting protein 1), followed by cg06938356, annotated to *COL23A1* (collagen type XXIII alpha 1 chain). We did not perform mediation analysis for the nine CpGs associated with both vitamin C treatment and wheeze, because our previous analysis suggests that FEF_25–75_ mediates the effect of vitamin C treatment on wheeze.

### Vitamin C-associated DMC genes are enriched among lung function and wheeze candidates

At the gene level, there were 477 genes containing one or more DMC associated with vitamin C treatment and FEF_25–75_, 253 genes that overlapped between vitamin C and wheeze DMCs, and 48 genes associated with treatment, FEF_25–75_, and wheeze at candidate significance of p < 0.001 (Additional file 2: Table S9). We examined protein–protein interactions using STRINGdb [41] for the 48 genes and identified “anatomical structure development” and “multicellular organism development” as biological processes significantly enriched within this list as well as enrichment for several human phenotypes which included “respiratory disease biomarkers,” “pulmonary function measurement,” and “FEV/FVC ratio” (Additional file 2: Table S9).

### Buccal DNAm signatures at 5 years are persistent from early life

**Fig. 3:**
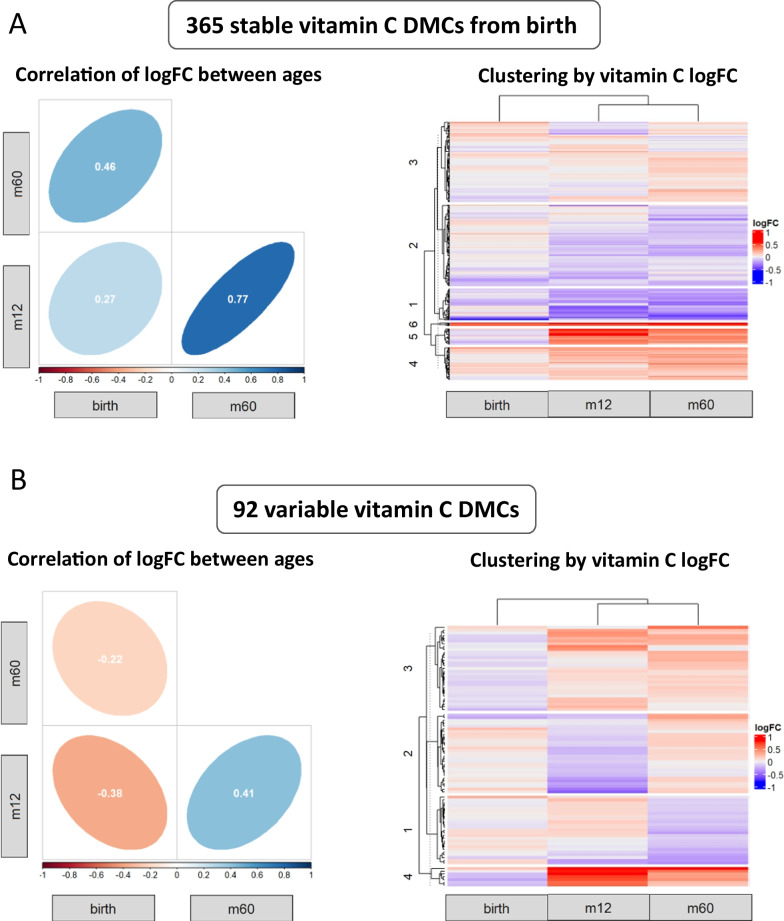
The majority of buccal DNAm signatures at 5 years are persistent from early life. Out of 457 vitamin C DMCs at 5 years, panel **A** shows the 365 CpGs with no significant interaction between time and treatment. The axes on the left panel are the vitamin C treatment effect sizes (logFC) at the indicated ages and the ellipses show the correlation (between 0 and 1) of treatment logFC between each pair of timepoints. The right panel is a heatmap of treatment effect sizes in these stable/persistent CpGs with hierarchical clustering. Panel **B** shows the remaining 92 CpGs with one or more significant interaction between treatment and time, repeating the correlation of logFC between ages in the left panel and heatmap clustering on the right

We next examined whether methylation profiles at vitamin C-associated DMCs observed at 5 years of age (m60) were established prenatally, or emerged with age **(**Fig. 3**).** We used buccal specimens collected from VCSIP RCT participants shortly after birth (m0; median = 2 days) and at ~ 12 months of age (m12; median = 371 days) and profiled epigenome-wide DNAm for a subset of buccal samples with sufficient DNA (*n* = 96 per age). Out of all buccal datasets passing QC, 37 subjects had DNAm data from all three ages (m0, m12, and m60) and spirometry data from the 5-year (m60) follow-up visit. We first tested the crude correlation of beta values between each pair of ages for each of the vitamin C treatment-associated DMCs. Out of 457 vitamin C FDR-DMCs, there were 47 vitamin C DMCs significantly correlated between m60 beta values and both birth and m12 beta values (Additional file 2: Table S10). We visually inspected the correlation between birth and 5-year-old beta values using scatterplots for each CpG and identified only one CpG that appeared to be genotype dependent (cg24245216), based on clusters of beta values near 0, 0.5, and 1, characteristic of a polymorphic site (Additional file 1: Fig. 2).

We performed the same correlation analysis across time for CpGs associated with FEF_25–75_ (*p* < 0.001). Out of 1,468 putative CpGs associated with 5-year-old lung function, 106 were highly consistent from birth until 5 years of age (Additional file 2: Table S10). Notably, 11 CpGs annotated to *PSORS1C3/ POUF41* and associated with 5-year-old FEF_25–75_ were highly correlated over time, suggesting this association was persistent since birth. Additional file 1: Fig. 3 depicts the correlation between raw beta values measured at birth versus at 5 years of age for one CpG in this region (cg11811828) (Additional file 1: Fig. 3A) and the correlation between cg11811828 beta values and FEF_25–75_ at 5 years of age (Additional file 1: Fig. 3B).

We next used a mixed-model-based approach to calculate the vitamin C treatment effect size (logFC) within this subset of 37 subjects at each age in the 457 FDR significant treatment DMCs, and to check for significant interactions between treatment and time (i.e., change in slope or direction of effect). Following our analysis of repeated methylation measures, we clustered DMCs based on interaction significance and logFC over time into two groups—(1) stable/persistent (i.e., without significant time interactions; *n* = 365 CpGs), and (2) variable (i.e., those with significant time*treatment interactions; *n* = 92 CpGs; Additional file 2: Table S11). Figure 3A (left) shows the Pearson correlation (between 0–1) of treatment logFC between each pair of timepoints/ages within the 365 CpGs persistent or stable from early life. Overall, the vitamin C treatment effect sizes (logFC) at 5 years were significantly correlated with logFC at 12 months of age and at birth (*R* = 0.77 and *R* = 0.46 respectively). A heatmap of treatment effect sizes in these stable/persistent CpGs suggests that the magnitude of difference between groups is increasing over time, similar to the observed effects of vitamin C treatment on lung function (Fig. 3A—right) [42]. This pattern is exemplified by one of the top FDR significant CpGs associated with vitamin C supplementation (cg08963132; *COL6A5*; Additional file 1: Fig. 4). In the 92 CpGs with significant interaction between vitamin C treatment and time, the correlation of vitamin C treatment effect sizes between m12 and m60 was positive (*R* = 0.41), while effect sizes at birth were negatively correlated with effect sizes at m12 and m60 (Fig. 3B—left). Additionally, hierarchical clustering of effect sizes in Fig. 3B (right) shows separation of the birth dataset from the m12 and m60 datasets, suggesting that methylation differences in these 92 CpGs emerged during the first year of life.

## Discussion

This study identifies differentially methylated loci in buccal DNA collected at 5 years of age from offspring of smokers randomized to vitamin C versus placebo during pregnancy [14]. We additionally tested whether methylation in buccal DNA is associated with respiratory outcomes in the same subjects and performed mediation analysis to identify potential epigenetic mechanisms for the observed effect of persistently improved lung function in the vitamin C exposed group. Lastly, we investigated whether the observed buccal DNAm associations from cross-sectional analysis at 5 years of age originated in the prenatal or early life period, or had emerged during childhood, using buccal DNAm from 37 subjects with samples collected near birth and near 12 months of age.

We identified 457 FDR significant vitamin C DMCs which were enriched for biological pathways related to growth and development, cellular stress, retinoic acid signaling, and cardiac hypertrophy, and the top DMCs were annotated to genes biologically relevant for lung development and function. *KCNK10* aka *TREK2* is a potassium channel member expressed in alveolar epithelial cells—and is a potential regulator of hyperoxia and mechanical ventilation induced lung injury in response to mechanical stretch [43, 44].

*COL6A5* (also known as *COL29A1* and *VWA4*) encodes collagen type 6 alpha 5 chain and is abundantly expressed in epithelial tissues such as the lung, skin, and colon. Several genome-wide association studies (GWAS) have previously linked this gene region to immune and respiratory related diseases including atopic dermatitis, asthma, atopy, and COPD [45–47]. Additional studies in animal models have suggested that collagen VI protein, comprised of three peptide chains which may include the α5 chain encoded by *COL6A5*, has a significant role in lung development [48]. Mice deficient for collagen VI protein exhibit simplified alveolar structure, similar to what is observed in bronchopulmonary dysplasia [49, 50].

*DDX39B* (also known as *BAT1*) encodes a helicase involved in maintaining genome stability [51]. Additionally, this gene is in the major histocompatibility complex region which contains multiple genes involved in immune signaling and function (e.g., TNFα and TNFβ), and knockdown of *BAT1* with antisense DNA increases production of cytokines such as TNFα, IL-1, and IL-6 [52]. Genetic polymorphisms in this locus have been linked to immune disease susceptibility such as atopic asthma [53], multiple sclerosis [54], and atopic dermatitis [55].

We examined the consistency of our results with those from previous studies using data from the EWAS Atlas [39]. Loci associated with vitamin C treatment were enriched for CpGs previously associated with atopy, immune disorders, and smoking [39]. Interestingly, one of the vitamin C DMCs previously associated with atopy in nasal cells (cg26575105; upstream of *FOXO3*) has also been associated with asthma in airway epithelial cells and with fractional exhaled nitric oxide in a separate nasal cell EWAS [18, 40, 56]. This gene encodes a member of the Forkhead transcription factor family, is important for DNA repair [57], is downregulated following various pro-fibrotic exposures in lung fibroblasts [58], and has been shown to modulate oxidative stress responses [59].

To identify candidate mediators for the observed effect of persistently improved lung function in the vitamin C exposed group, we also performed an EWAS of lung function at 5 years of age, represented by FEF_25–75_ in this study, and performed mediation analysis in 62 CpGs associated with both vitamin C treatment and lung function. The largest mediation effect size was observed for *JAKMIP1* (janus kinase and microtubule interacting protein 1), a gene dysregulated in neurodevelopmental disorders [60] which contains a maternally imprinted DMR that may impact fetal growth [61]. Although the top loci associated with lung function were not associated with vitamin C treatment, we identified a highly significant lung function DMR near *POU5F1* and *PSORS1CS. PSORS1CS* promoter activity is responsive to the glucocorticoid dexamethasone and in turn modulates *POU5F1/OCT4* expression, which is proposed to explain association of this psoriasis susceptibility candidate with various immune related diseases [62] and may also explain the observed association with lung function. Additionally, *FAM181A* was positively associated with FEF_25–75_ in this study, while methylation at *FAM181A* is decreased in blood from severe asthmatics relative to controls and increased in peripheral blood from infants whose mothers were asthmatics during pregnancy [63, 64].

Our longitudinal analysis of methylation in a subset of participants with buccal DNA collected at birth and near 1 year of age demonstrated that most loci associated with prenatal vitamin C and/or lung function at 5 years of age were established in utero, rather than emerging later in life. The effect size between vitamin C supplemented and placebo groups was often larger at 5 years of age than in early life, and in the same direction. This pattern parallels what we have observed in measurements of lung function in this cohort, in which differences in forced expiratory flows also increase over time [42]. This further supports the linkage between methylation changes and pulmonary function changes suggested by the mediation analysis. For top lung function associated candidates, the methylation profiles were remarkably stable from birth until 5 years of age. In support of this result, a previous study examined association of methylation in cord blood DNA with lung function from 8 to 24 years, and noted sex-specific stable patterns of DNAm from birth associated with lung function development [65].

Our findings suggest that DNA methylation in airway epithelium, represented here by buccal epithelium, may mediate some of the effects of supplemental vitamin C to pregnant smokers on offspring respiratory outcomes. Our epigenome-wide DNAm findings suggest multiple potential pathways that could relate to alterations in airway structure, function, reactivity, and inflammation. Although we did not identify any genome-wide DMCs for wheeze, the phenotype of recurrent wheeze (often diagnosed as asthma) encompasses a variety of pathophysiologic mechanisms with distinct cellular and molecular signatures, referred to as endotypes [66]. More extensive endotyping of this unique cohort is necessary to better understand the relationships between DNAm and phenotype/endotype within distinct sub-populations.

Unique strengths of our study are our ability to combine epigenome-wide buccal DNAm analysis with direct measurements of lung function in childhood in a majority smoking population randomized to an in utero intervention. For this cohort, we have collected extensive exposure (e.g., serial measures of cotinine, hair nicotine, and ascorbic acid) and outcome measurements at multiple timepoints throughout pregnancy and during early childhood. In a smaller number of subjects, we have collected DNA samples longitudinally beginning at birth for analysis of methylation trajectories associated with lung function trajectories. We are continuing to follow this cohort for respiratory phenotyping and collection of biospecimens to determine the persistence of the effects of vitamin C supplementation during pregnancy on methylation and later health outcomes.

While multiple studies have examined epigenome-wide DNAm in buccal cells in the context of current adult smoking and related pathologies such as COPD and lung cancer [67, 68], few studies have been performed in this tissue for prenatal smoke exposure [29, 35, 69], and to our knowledge none epigenome-wide. However, several studies have observed that nasal cell DNAm is associated with asthma, lung function, and other airway diseases and phenotypes [40, 60, 70–73]. An area of future study for this cohort as they age will be to examine epigenome-wide DNAm in buccal versus nasal epithelium and to determine if additional mechanistic information can be obtained with the more invasive sample technique.

A limitation to our study is our sample size, which is underpowered compared to large population-based studies to detect small differences in methylation at individual CpGs. As we anticipated that differences in methylation associated with vitamin C supplementation and/or respiratory measures would be small relative to other exposures, such as those observed with smoking, we considered a nominal significance threshold of *p* < 0.001 to identify candidates for pathway analysis and for future validation. We also performed DMR analysis to identify regions with correlated methylation structure that are more likely to impact transcriptional regulation than single CpGs, depending on the genomic context of regions.

Additional limitations are that we had fewer buccal samples from non-smokers as a reference of DNAm in healthy subjects, since our study was established as a randomized trial of smokers. In addition, the DNA quality was not adequate for all samples from the offspring of randomized smokers, therefore these results represent only 158/192 subjects who completed study visits at 5 years of age. Considering the demographics of participants in our study and the additional challenge of the COVID-19 pandemic during the study period, our overall cohort retention was excellent.

We were unable to perform analysis of gene expression in these buccal samples to determine whether methylation differences had a functional impact; however, we plan to include this in future analysis using nasal epithelium. Lastly, the single CpG mediator analysis approach does not consider the combined role that DNA methylation at multiple loci plays in the association between prenatal vitamin C exposure and FEF_25–75_ at 5 years of age, nor does it consider epigenetic regulation of gene expression. Although these findings are suggestive, in that we used a less stringent threshold for DMC candidates; they are strengthened by our downstream analyses demonstrating significant mediation of treatment effects, by consistency with prior EWAS and enrichment for loci associated with relevant phenotypes, by repeated measures analysis in a subset of subjects, and by biological plausibility for top differentially methylated loci to potentially impact lung development and function.

## Conclusions

This epigenome-wide analysis of buccal DNA methylation within a randomized clinical trial population of pregnant smokers receiving vitamin C supplements or placebo identifies significant differences in methylation at 5 years of age that likely originated in utero. Critically, some of the treatment associated CpGs were also associated with lung function measured at 5 years of age, and differences in methylation increased over time in these CpGs just as offspring forced expiratory flows increased over time [16]. These findings suggest the potential for DNA methylation in airway tissues to mediate some of the effects of vitamin C supplementation on lung function and respiratory health in offspring exposed to maternal smoking in utero.

## Materials and methods

### Study design

This study was a follow-up analysis of our multi-center, double-blind, placebo-controlled RCT that demonstrated improved airway function at 3, 12, and 60 months of age in offspring whose mothers were unable to quit cigarette smoking and were randomized to supplemental vitamin C (500 mg/day) versus placebo during pregnancy [53, 54, 58]. A total of 252 patients were randomized (1 excluded for protocol violation; 125 allocated to vitamin C; 126 allocated to placebo) and of those subjects 192 (93 vitamin C; 99 placebo) had acceptable PFTs from in person visits at 5 years (60 months) of age. For the current study, we included all subjects with sufficient buccal DNA quality and pulmonary function testing performed at the 5-year-old visit (*n* = 158; Additional file 1: Fig. 1).

### Study population

The parent RCT recruited women with singleton pregnancies (≥ 15 years old; < 23 weeks gestation) with a history of current cigarette smoking and documented refusal/inability to quit. Women were randomized to receive vitamin C (500 mg/day) versus placebo after a successful run-in trial for medication compliance that required 75% adherence and return for follow-up within 7 to 21 days. A standard prenatal vitamin containing the minimum daily requirement (MDR) of vitamin C was also provided to all participants. Randomization to vitamin C or placebo was blocked in rotations of two and four subjects, and stratified by gestational age at randomization (≤ 18 versus > 18 weeks) and site (Oregon Health & Science University [OHSU], Portland, Oregon; PeaceHealth Southwest Washington Medical Center [SWW], Vancouver, Washington; Indiana University [IU], Indianapolis, Indiana). The RCT was approved by each site’s Institutional Review Board and monitored by an NIH appointed Data Safety Monitoring Board. We obtained written informed consent from all subjects prior to enrollment [14].

### Statistical analysis of patient demographics

We summarize patient characteristics in Table 1. Continuous variables were summarized using mean and standard deviation and categorical variables were summarized in percentages after excluding missing values. We tested for differences between groups using Pearson's Chi-squared test for categorical variables, one-way analysis of variance for normally distributed continuous variables (i.e., regular ANOVA). Non-normally distributed variables were assessed for group differences using Fisher’s exact test for categorical data and Kruskal–Wallis rank sum test for continuous data.

### Respiratory phenotyping

We measured airway function and wheeze in infants and children born to smoking participants in the RCT as described previously [16]. Spirometry was performed at 5 years of age using a model 6800 spirometer (Vitalograph) and adhering to American Thoracic Society (ATS)/ European Respiratory Society (ERS) acceptance guidelines. A standardized respiratory questionnaire (RQ) was administered quarterly to the child’s parent or primary caretaker. Wheeze was defined a priori as a positive response to any of the following: parental report of wheeze, healthcare professional diagnosis of wheeze or asthma, or any bronchodilator or steroid use. Only children with one or more RQs completed after their fourth birthday were included in the analysis.

### Collection and processing of DNAm

Epigenome-wide methylation profiles were measured in DNA from buccal epithelial cells using the MethylationEPIC BeadChip (Illumina, San Diego, California) at the Fred Hutchinson Cancer Genomics Resource (Seattle, WA). See Additional file 1 for details of buccal cell collection, DNA extraction, and DNA methylation acquisition. Data normalization and QC were performed using ChAMP [74]: non-CpG probes, probes with a beadcount < 3 in at least 5% of samples, probes annotated to SNPs [75], probes with a detection *p* value > 0.01 in one or more samples, cross-hybridizing probes [76], and probes on X/Y chromosomes were removed and remaining probes (*n* = 746,421) were normalized via functional normalization. We used the EpiDish R package to estimate proportions of epithelial cells, fibroblasts, and total immune cells in our buccal DNA samples [77].

### Correction for technical factors and estimation of latent covariates

Principal component analysis (PCA) and ChampSVD were used to identify correlation with potential covariates. Significant sources of variation unrelated to study design were identified in a step-wise fashion. First, we identified cell composition as the top source of variation and used linear regression to calculate residual variance after adjustment for estimated proportion of epithelial cells. Second, we used Combat to regress out variation from technical variables (slide, array, and DNA concentration) [78]. We calculated methylation values (*M* values) as the logit2 combat adjusted beta values. In order to adjust for unmeasured confounding, we used the CorrConf package to determine the number of latent covariates separately for each analysis (after protecting dependent variable effects) and to generate a new design matrix which includes latent variables [79].

### Analysis of differentially methylated CpGs (DMCs) and regions (DMRs)

To assess methylation differences in buccal DNA, we used *M* values in linear models adjusted for gestational age at randomization (≤ 18 versus > 18 weeks), site, sex, race (white versus non-white), cell composition, and latent covariates. We performed sensitivity analyses without cell proportions and latent covariates in the models to determine whether DNA methylation differences between groups were confounded by treatment related changes in cell composition. In the models for lung function, we added length at PFT as a continuous variable.

Differentially methylated CpGs (DMCs) were assessed using the Benjamini–Hochberg procedure (FDR) < 5%. Differentially methylated regions (DMRs) were assessed using DMRcate with recommended settings for array data [80] and the same models used for DMC analysis. DMRs were considered significant if there were 2 or more DMCs within a 1000 bp span and *p* < 0.001 after FDR correction. Because there were few FDR significant DMCs in our analyses for FEF_25–75_ and for wheeze, we used less stringent criteria to obtain candidate DMRs (pcutoff = 0.2), but required the same level of significance (FDR *p* < 0.001) in filtering the resulting DMRs. For downstream enrichment analysis, we used gene and region annotation provided for each probe in the Illumina HumanMethylationEPIC annotation file. Intergenic CpGs were mapped to the nearest proximal gene using the chromosome and positions (GrCh37/hg19), using the *GenomicRanges* package in R.

### Comparison of DMCs with previous EWAS

From the EWAS Atlas (https://ngdc.cncb.ac.cn/ewas/downloads) we downloaded 643,805 previous EWAS associations, along with study and cohort information, from over 1000 publications [39]. We used the online EWAS Toolkit to calculate enrichment for traits, GO terms, etc., and mapped our DMCs to previous studies for easy reference in Additional file 2: Table S3.

### Overlap of treatment and lung function DMCs and mediation analysis

We used the VennDiagram *R* package to visualize the overlap between treatment DMCs and outcome DMCs (*p* < 0.001) and to perform the hypergeometric test at the probe level and gene level. We then evaluated whether any proportion of the association between vitamin C supplementation and FEF_25–75_ is mediated by DNAm. We considered as candidates for mediation analysis only those CpGs with a significant association with both vitamin C supplementation and FEF_25–75_ (both at *p* < 0.001). We used the mediation R package [81] with 500 Monte Carlo simulations per CpG to estimate the direct effect between vitamin C treatment and FEF_25–75_ and the effect mediated through DNAm (vitamin C → DNAm → FEF_25–75_). Our mediation models included race, sex, site, GA_strata, PFT height, and Epi proportions as covariates. We considered mediation significant if the FDR adjusted average causal mediation effect (ACME) *p* value was less than 0.05.

### Functional enrichment analysis of biological pathways and phenotypes

We used the EWAS Toolkit [39] to test for enrichment of EWAS traits, gene ontology (GO), and KEGG pathways among FDR significant DMCs based on probe ID. The GO and KEGG enrichment analysis is implemented based on the gometh function in the missMethyl package [36] which calculates enrichment after accounting for bias due to either (1) a greater number of probes per gene covered in the array dataset, or (2) CpGs which are annotated to multiple genes. As background for enrichment, we used all genes annotated to the EPIC platform. We used the STRING database [41] to look at protein–protein interaction networks, enriched GO terms and diseases among the 48 overlapping genes in Additional file 2: Table S9.

### Longitudinal analysis of methylation

Out of all buccal datasets collected passing QC, 37 subjects had methylation available for all 3 ages (m0, m12, and m60) and spirometry data at the 5 year follow-up visit. We performed Pearson correlation for each of the 457 vitamin C FDR-DMCs and the 1,468 candidate (*p* < 0.001) DMCs associated with FEF_25–75_ using the m60 vs m12 beta values, and separately the m60 vs the m0 beta values. *P* values were corrected for multiple testing using the Benjamini & Hochberg ("BH" or its alias "fdr") method to control for family-wise error.

We next performed mixed-model linear regression of repeated measures over time to examine the interaction between treatment effects and age at DNA collection. We estimated the average correlation between the 37 subjects and included subject as a blocking variable, adjusting for biological sex at birth. We did not adjust for additional covariates in the repeated measures analysis due to limited sample size and correlated covariates across time. We assessed the logFC for vitamin C treatment at each age of collection (m0, m12, and m60) and the *p* values for interaction (m60 vs m0, m60 vs m12, and m12 vs m0). If no significant interaction (*p* < 0.05) for any interval was observed, and the logFC was in the same direction and treatment *p* < 0.05, DMCs were considered stable or persistent since birth.

### Assessment of methylation patterns indicating genetic variance

We visually inspected the correlation between birth and 5-year-old beta values for potential genotype effects using a scatterplot for each significant CpG. We inferred that methylation was genotype dependent if there was a methylation pattern characteristic of polymorphic sites (e.g., *β* values fall into two or three levels, with gaps in between, when plotted on a continuous scale of 0–1). We additionally used the R package “MethylToSNP” which performs an automated analysis of methylation data distributions and gaps for the detection of SNP-like patterns [82].

### Supplementary Information

**Additional file 1: Fig. S1** Consort diagram and EWAS overview. Out of 252 pregnant smokers randomized to vitamin C or placebo as part of our VCSIP RCT, 243 offspring were delivered and 213 were re-consented into follow-up through 5 years of age. Of 192 subjects with technically acceptable spirometry at 5 years of age, 158 had sufficient quality buccal DNA for epigenome-wide methylation analysis (EWAS; n= 80 placebo, 78 vitamin C). We first performed an EWAS for vitamin C vs. placebo, followed by EWAS for FEF25-75 and current wheeze.** Fig. S2** Example of scatterplot for inspection of genotype effect. We inferred that methylation was genotype dependent if there was a methylation pattern characteristic of polymorphic sites (e.g.* β*-values fall into two or three levels, with gaps in between, when plotted on a continuous scale of 0–1).** Fig. S3** Persistent association of PSORS1C3 with FEF25-75. Among CpGs persistent over time, we highlight the correlation of one CpG (cg11811828) measured at birth versus at 5 years of age (A) and the correlation between beta values and FEF25-75 at 5 years of age (B).**Fig. S4** Longitudinal plots for COL6A5. In panel** A**, we show the smoothed mean and confidence interval for each treatment for cg08963132 over time. In panel** B**, we show the correlation between raw beta values measured at birth and beta values measured at 5 years of age. Supplemental Methods.

**Additional file 2 : Table S1. **DMCs associated with vitamin C at FDR significance.** Table S2** DMRs associated with vitamin C at FDR significance.** Table S3** Vitamin C associated probes identified in previous EWAS using the EWAS Atlas.** Table S4** DMCs associated with FEF25-75 (p<0.001).** Table S5** Candidate DMRs associated with FEF25-75.**Table S6**. DMCs associated with wheeze (p<0.001).** Table S7** Candidate DMRs associated with wheeze.** Table S8** Mediation results for 62 CpGs associated with both Vitamin C supplementation and FEF25-75 at p<0.001.** Table S9**. Enrichment of biological pathways and phenotypes in 48 genes overlapping in the 3 EWAS.** Table S10** Overlap of FDR significant correlation of 5-year buccal DMC beta values with DNA collected at birth and near 12 months of age.** Table S11** Longitudinal analysis of buccal methylation in 37 patients with samples at m0, m12, and m60.

## Data Availability

The raw and processed DNA methylation used and/or analyzed during the current study are publicly accessible through NCBI Gene Expression Omnibus (GEO) via accession series GSE253158.

## Abbreviations

MSDP: Maternal smoking during pregnancy
CpG: Cytosine–guanine dinucleotide
DNAm: DNA methylation
DMC: Differentially methylated CpG
DMR: Differentially methylated region
EWAS: Epigenome-wide association study
RCT: Randomized clinical trial
VCSIP: Vitamin C supplementation in pregnancy
IUGR: Intrauterine growth restriction
FDR: False discovery rate
PFT: Pulmonary function test
FEF: Forced expiratory flow
FEF_25–75_: Forced expiratory flow between 25 and 75% of expired volume (also known as MMEF maximal mid-expiratory flow)
GO: Gene ontology
GEO: Gene expression omnibus
