# Correction: Improvements in lung function following vitamin C supplementation to pregnant smokers are associated with buccal DNA methylation at 5 years of age

**DOI:** 10.1186/s13148-024-01664-4

**Published:** 2024-04-25

**Authors:** Lyndsey E. Shorey-Kendrick, Cindy T. McEvoy, Kristin Milner, Julia Harris, Julie Brownsberger, Robert S. Tepper, Byung Park, Lina Gao, Annette Vu, Cynthia D. Morris, Eliot R. Spindel

**Affiliations:** 1 Division of Neuroscience, Oregon National Primate Research Center, Oregon Health and Science University, Beaverton, OR 97006 USA; 2 Department of Pediatrics, Pape Pediatric Research Institute, Oregon Health and Science University, Portland, OR USA; 3 Department of Pediatrics, Oregon Health and Science University, Portland, OR USA; 4 Department of Pediatrics, Herman B Wells Center for Pediatric Research, Indiana University School of Medicine, Indianapolis, IN USA; 5 Biostatistics Shared Resources, Knight Cancer Institute, Bioinformatics and Biostatistics Core, Oregon National Primate Research Center, Oregon Health and Science University, Portland State University School of Public Health, Portland, OR USA; 6 Oregon Clinical and Translational Research Institute, Oregon Health and Science; Department of Medical Informatics and Clinical Epidemiology, Oregon Health and Science University, Portland, OR USA

**Correction to: Clinical Epigenetics (2024) 16:35** 10.1186/s13148-024-01644-8

Following publication of the original article [1], the authors noticed that the NCBI Gene Expression Omnibus (GEO) accession series has been incorrectly listed as GSE253158 within the “Availability of data and materials section”. The correct GEO series for this work is GSE252169.
