# Supplementary material for: Improvements in lung function following vitamin C supplementation to pregnant smokers are associated with buccal DNA methylation at 5 years of age
Source: Clin Epigenetics. 2024 Feb 27;16:35. doi: 10.1186/s13148-024-01644-8 (PMC10900729; doi:10.1186/s13148-024-01644-8)
Supplement: Supplementary file 1 — Additional file 1: Fig. S1 Consort diagram and EWAS overview. Out of 252 pregnant smokers randomized to vitamin C or placebo as part of our VCSIP RCT, 243 offspring were delivered and 213 were re-consented into follow-up through 5 years of age. Of 192 subjects with technically acceptable spirometry at 5 years of age, 158 had sufficient quality buccal DNA for epigenome-wide methylation analysis (EWAS; n= 80 placebo, 78 vitamin C). We first performed an EWAS for vitamin C vs. placebo, followed by EWAS for FEF25-75 and current wheeze. Fig. S2 Example of scatterplot for inspection of genotype effect. We inferred that methylation was genotype dependent if there was a methylation pattern characteristic of polymorphic sites (e.g. β-values fall into two or three levels, with gaps in between, when plotted on a continuous scale of 0–1). Fig. S3 Persistent association of PSORS1C3 with FEF25-75. Among CpGs persistent over time, we highlight the correlation of one CpG (cg11811828) measured at birth versus at 5 years of age (A) and the correlation between beta values and FEF25-75 at 5 years of age (B).Fig. S4 Longitudinal plots for COL6A5. In panel A, we show the smoothed mean and confidence interval for each treatment for cg08963132 over time. In panel B, we show the correlation between raw beta values measured at birth and beta values measured at 5 years of age. Supplemental Methods. [file 13148_2024_1644_MOESM1_ESM.pdf]

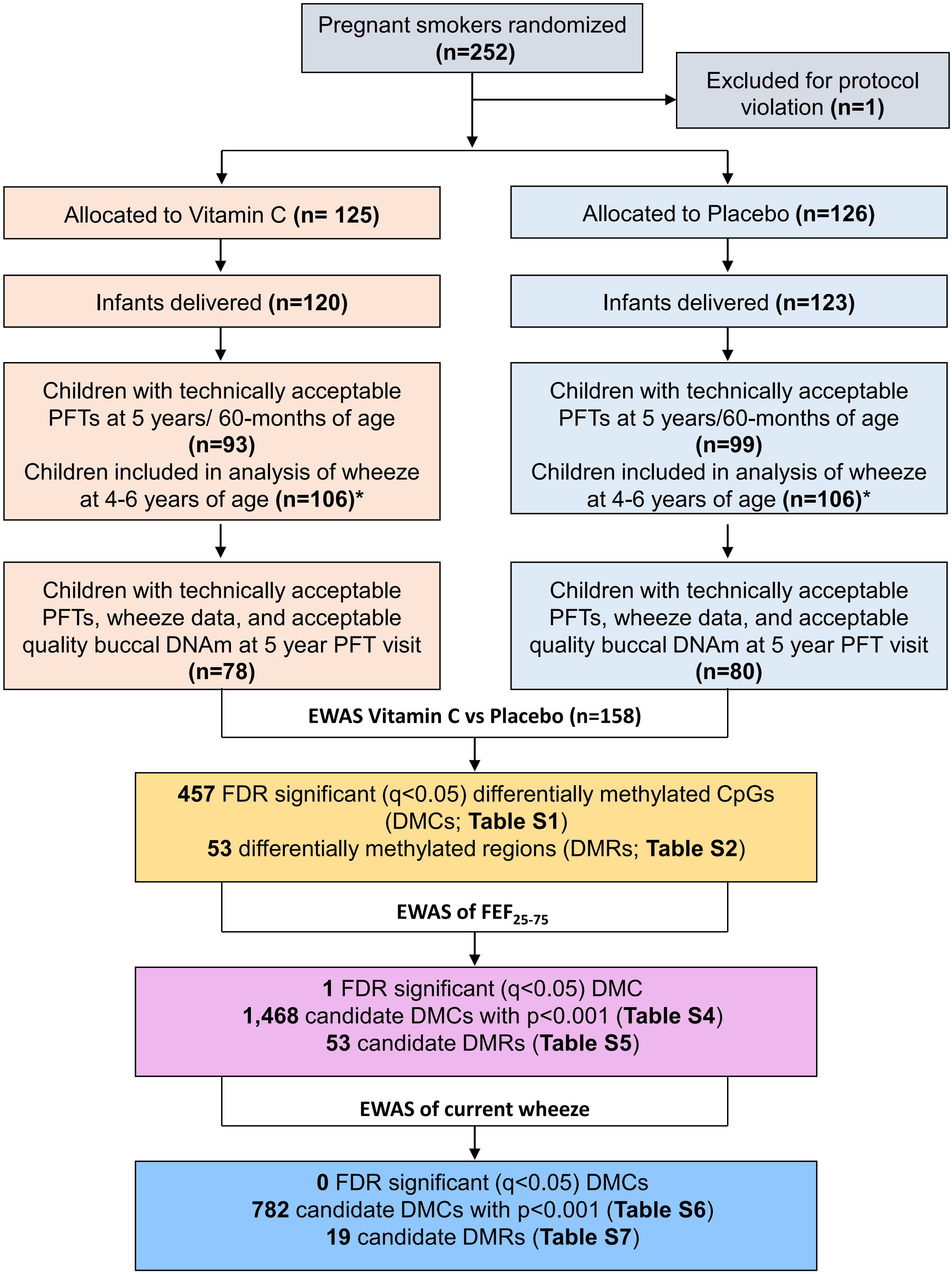
Pregnant smokers randomized (n=252)
Excluded for protocol violation (n=1)
Allocated to Vitamin C (n= 125)
Allocated to Placebo (n=126)
Infants delivered (n=120)
Infants delivered (n=123)
Children with technically acceptable PFTs at 5 years/ 60-months of age (n=93) Children included in analysis of wheeze at 4-6 years of age (n=106)*
Children with technically acceptable PFTs at 5 years/60-months of age (n=99) Children included in analysis of wheeze at 4-6 years of age (n=106)*
Children with technically acceptable PFTs, wheeze data, and acceptable quality buccal DNAm at 5 year PFT visit (n=78)
Children with technically acceptable PFTs, wheeze data, and acceptable quality buccal DNAm at 5 year PFT visit (n=80)
EWAS Vitamin C vs Placebo (n=158)
457 FDR significant (q<0.05) differentially methylated CpGs (DMCs; Table S1)
53 differentially methylated regions (DMRs; Table S2)
EWAS of FEF_{25-75}
1 FDR significant (q<0.05) DMC
1,468 candidate DMCs with p<0.001 (Table S4)
53 candidate DMRs (Table S5)
EWAS of current wheeze
0 FDR significant (q<0.05) DMCs
782 candidate DMCs with p<0.001 (Table S6)
19 candidate DMRs (Table S7)

Example of genotype inspection

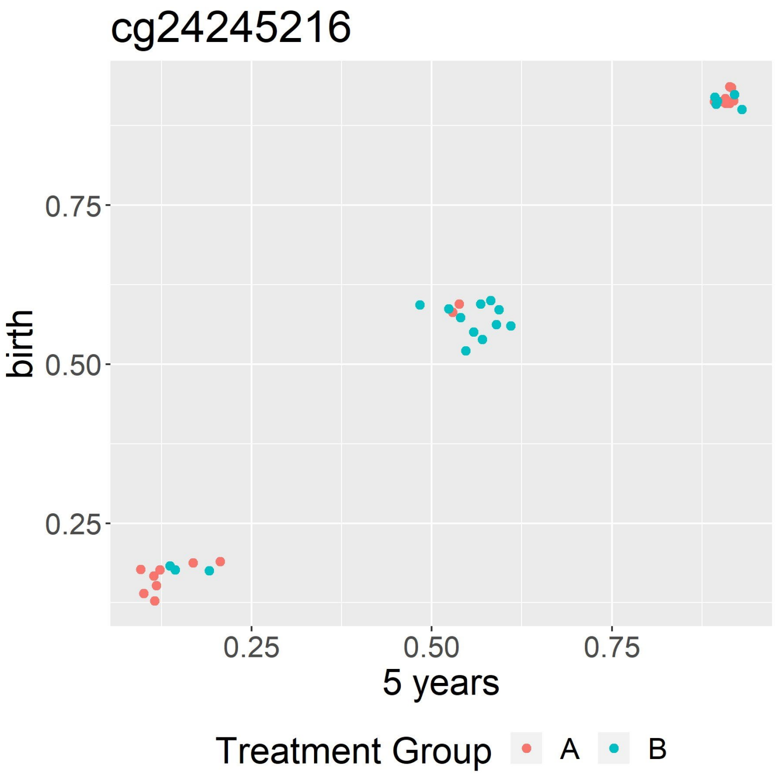

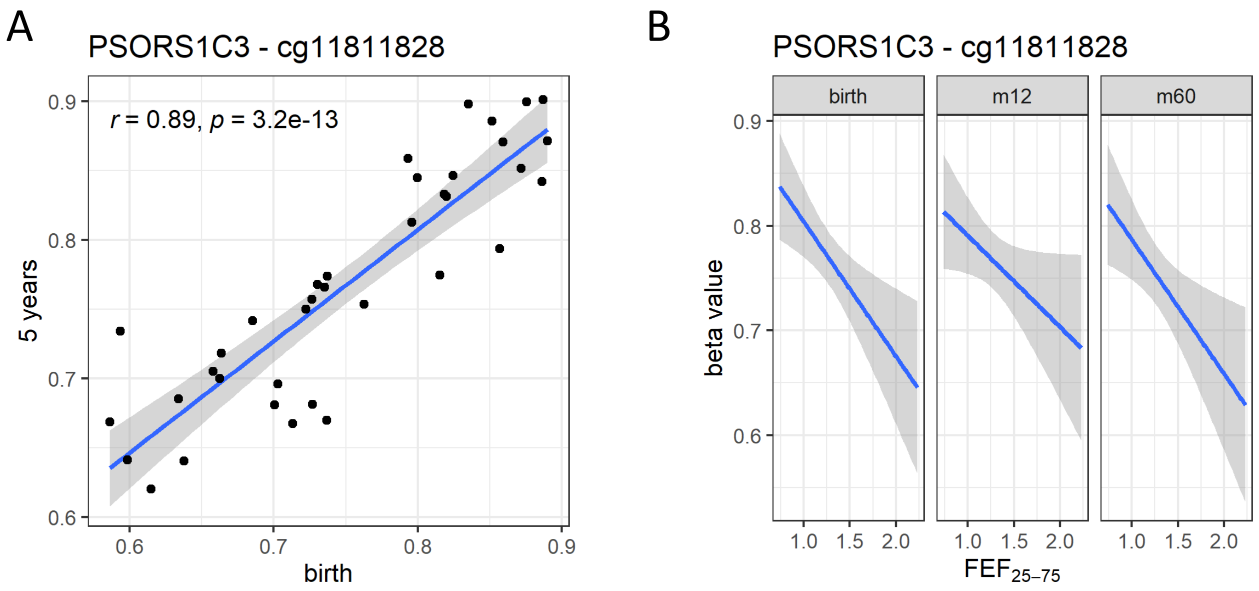
A
PSORS1C3 - cg11811828
r = 0.89, p = 3.2e-13
5 years
birth
0.6
0.7
0.8
0.9
B
PSORS1C3 - cg11811828
birth
m12
m60
beta value
FEF25–75
1.0
1.5
2.0

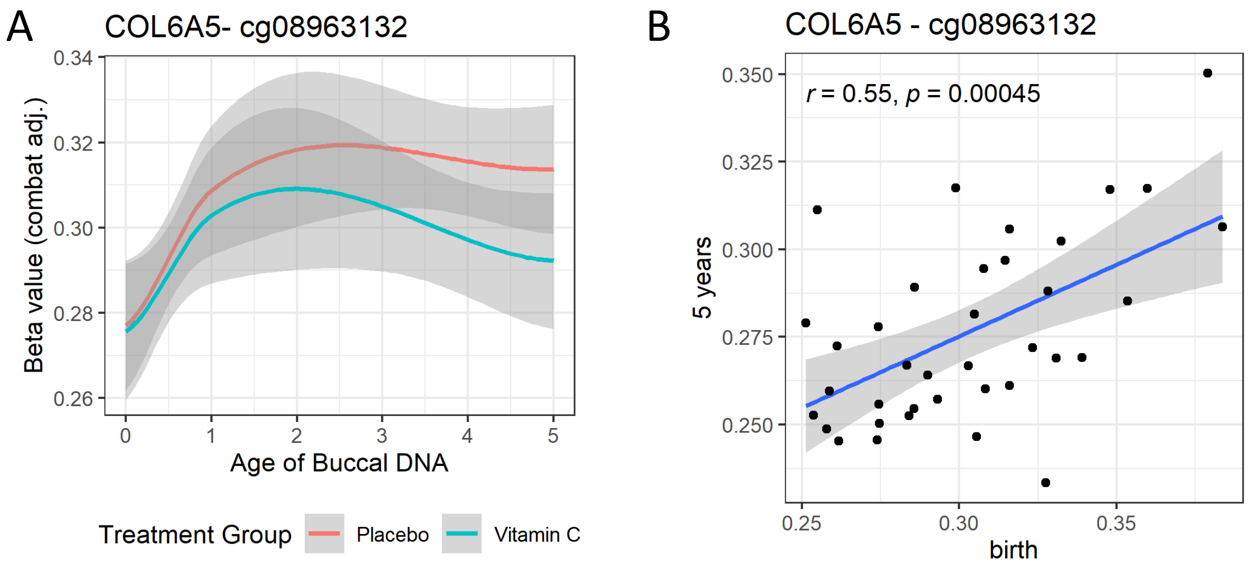
A
COL6A5- cg08963132
Beta value (combat adj.)
0.34
0.32
0.30
0.28
0.26
0
1
2
3
4
5
Age of Buccal DNA
Treatment Group
Placebo
Vitamin C
B
COL6A5 - cg08963132
r = 0.55, p = 0.00045
5 years
0.350
0.325
0.300
0.275
0.250
0.25
0.30
0.35
birth

Supplemental Methods:

Collection of buccal samples

Patients were instructed not to eat or drink at least 30 minutes before buccal sample collection. Buccal DNA was collected using 4 Sterile HydraFlock® Large Tip Flocked Swabs (Puritan 253406H). Each swab was applied to the inside of the child's cheek and rolled up and down for 30-60 seconds, making certain to roll the swab over the entire inner cheek and upper gum. Swabs were placed together in a 15 ml conical tube containing 1600ul of 1X Saliva Lysis Buffer (SLB) before capping tube and vortexing for 10 seconds. The swabs were frozen upright in SLB at -20°C within 1 week from sampling and stored until DNA extraction.

<u>2X Saliva Lysis Buffer</u>
0.3 M TRIS-HCl
0.67 M urea
0.67 M NaOAc
0.6% sodium dodecyl sulfate
3.3 mM EDTA
30% ethanol

Extraction of buccal DNA

Buccal DNA was extracted using the Maxwell® 16 Instrument (Promega AS3000) and Maxwell® 16 Blood DNA Extraction Kit (Promega AS1010). Proteinase K (Promega 20mg/ml) was added to 15ml conicals containing 4 swabs and ~1.6ml SLB. Tubes were vortexed to collect SLB at bottom. Tubes were incubated in s 56°C water bath for 1 hr and then vortexed. Lysate (800ul) was added to well #7 of the Maxwell® 16 Blood DNA cartridge. The remaining lysate was stored for a second DNA extraction. Alcohol Wash Buffer (2 parts Milli-Q water, 1 part 100% EtOH, 1 part 100% isopropanol) was added to wells #4, #5 and #6. Cartridges were processed using the Buffy Coat Protocol on the

Maxwell® 16 Instrument and eluted in 400ul elution buffer. Purity was measured by spectrophotometer and concentration measured with the Quant-iT PicoGreen dsDNA Assay (Invitrogen® P7589).

Quantification of DNAm

Buccal DNAm was assessed on the MethylationEPIC BeadChips, measuring over 850,000 CpGs at a nucleotide resolution, at the Fred Hutchinson Cancer Research Center Genomics Resource. In brief, up to 500ng of DNA was bisulfite converted using the EZ DNA Methylation Kit (Zymo Research) following the Illumina-specified instructions. Converted DNAs were applied to Illumina Infinium Methylation EPIC 8-Sample Beadchips following the Infinium HD Methylation 15019521v01 protocol. Processed BeadChips were scanned using the Illumina iScan+ with ICS v3.3.28 and intensity data was extracted with Illumina GenomeStudio software (GenomeStudio v2011.1 with Methylation Analysis Module v1.9.0).
